# A compact-rigid multi-analyser for energy and angle filtering of high-resolution X-ray experiments. Part 1. Principles and implementation

**DOI:** 10.1107/S160057752201116X

**Published:** 2023-01-01

**Authors:** A. Prat, J.-L. Hodeau

**Affiliations:** a Institut Néel CNRS-UGA, 25 Avenue des Martyrs, 38042 Grenoble, France; University of Malaga, Spain

**Keywords:** high-resolution powder diffraction, multi-analyser diffraction filtering, heterogeneous materials, high-resolution experiments, compact-rigid multi-analyser

## Abstract

Diffraction and spectroscopy instruments using a filtering process with several analyser crystals have existed for about 30 years at synchrotron radiation sources, but they are difficult to use on laboratory sources. Several diffraction multi-filtering systems for powder diffraction experiments have been studied and optimized, in order to show the relevance, simplicity and efficiency of their implementation. Optical filter systems containing one or many diffracting elements, precisely positioned in a rigid manner on a logarithmic spiral surface and having a stability that allows high resolution and high sensitivity to powder diffraction experiments, have been developed. After having tested prototypes with various geometries, we present in particular the realization of a small rigid-compact multi-analyser comb that allows 20–50 measurements on synchrotron radiation sources to be filtered in parallel, but also and especially that can be adapted on laboratory X-ray sources (Ag *K*α_1_) to increase by an order of magnitude the intensities and resolutions of the measurements. Such a rigid-compact multi-analyser block can advantageously be associated with ‘photon-counting’ 1D and 2D detectors in order to drastically improve the detection thresholds of powder diffraction measurements to better than 0.1%, which allows the detection/quantification/analysis of minor phases in studies of ‘real’ complex materials.

## Abstract 2

Principles and implementation of a compact-rigid multi-analyser for energy and angle filtering of high-resolution X-ray experiments are presented. Several systems containing one or many diffracting elements positioned in a rigid manner on a logarithmic spiral surface are discussed and developed in order to detect/analyze minor phases of complex materials.

## 1. Introduction

For X-ray structural analysis, the development of position-sensitive 1D or 2D detectors increases the statistics of diffraction, fluorescence and imaging studies, allowing a considerable reduction in measurement times compared with experiments using angular scanning point detectors. Unfortunately the instrumental angular resolution of these 1D or 2D detectors, which is determined by both their pixel size and the sample-to-detector distance, can be quite low and there are few ways to filter out their external noise both in energy and in direction. This limitation is a major handicap and moreover it is not possible to fully exploit the gain given by the new 2D ‘photon-counting’ detectors which have minimal intrinsic noise. It is therefore essential to develop tools to perform an efficient filtering of 1D or 2D data in order to overcome this latter bottleneck and to further improve the signal-to-noise ratio of experiments, thus to make structural analyses much more accurate and selective.

In experiments using angular scanning point detectors (0D detectors), this filtering is often achieved by inserting a crystal analyser (or back-side monochromator) between the sample and the detector, which only detects the ‘good’ photons. This single-crystal-analyser filter is based on diffraction which selects a precise angle Θ_A_ of the beam from the sample and acts as an energy band filter (depending on the structure of the crystal analyser and the *d*_*hkl*_ spacing of the *hkl* planes used) and as a beam divergence filter (depending on its small aperture slits). This diffraction filtering has a high selectivity which is related to the sharpness of the angle Θ_A_ between the beam coming from the sample and the *hkl* crystal planes of the analyser. However, such a diffractive analyser filter has a major limitation due to its low efficiency and the imperative stability of its alignment. These limitations mean that it is almost exclusively used in instruments using very intense sources generated by synchrotron radiation (SR), where many studies have been carried out in the past to understand and optimize this diffraction filtering. With a single-crystal analyser, we have a directional selectivity that allows us to select the beams coming from only a given part of the sample and to determine precisely their emission angles by the sample; moreover, such a diffractive analyser allows us to select the energy of the beams scattered by the sample and/or to suppress the fluorescence radiation. Such a system has been developed for high-resolution diffraction and spectroscopy experiments where (i) for spectroscopy experiments, the important data is the energy of the beam transmitted by the sample that is accurately determined and (ii) for diffraction experiments, the important data is the angle 2Θ of the beam diffracted/scattered by the sample that is accurately determined.

As the key element of a diffractive analyser filter is a perfect/mosaic single crystal, the development of a multiple analyser system using diffraction (MAD-filter), which can be used with 1D or 2D detectors, requires a corresponding increase in the number of perfect/mosaic analyser crystals that must all have the correct orientation to diffract at the same angle Θ_A_! Therefore, we need to develop a stable MAD-filter having many orientations for each of its ‘crystal areas’ that perfectly match this angle Θ_A_. Following the previous studies reported in the next section, we study and compare the gains and limitations of different MAD-filters having different geometrical configurations in order to optimize them, and we propose to support all these diffracting crystal areas on a surface having a logarithmic spiral shape. Then we present and discuss several possibilities to increase the efficiency of such logarithmic spiral curvatures (‘LogSpiral’ curvatures) by using either (i) curved single-crystal analysers diffracting in Bragg reflection mode, or (ii) a curved thin crystal analyser diffracting in Laue transmission mode, or (iii) planar single-crystal analyser arrays diffracting in Bragg reflection mode. Finally, we compare a few compact fixed/rigid filtering devices using an analyser array associated with a Soller collimator and a 2D detector. These studies show that such systems could also be used to increase the resolution and flux of diffraction experiments using laboratory sources.

## 2. History

The principle and advantages of diffraction filtering using an analyser crystal between the sample under study and the detector was developed in the 1980s and 1990s in research facilities using SR, to enable powder diffraction experiments (Cox *et al.*, 1983 ▸; Hastings *et al.*, 1984 ▸). By optimizing the optical geometries of the instrument and the SR beamline (Sparks *et al.*, 1982 ▸; Batterman & Berman, 1983 ▸; Ferrer *et al.*, 1992 ▸; Hazemann *et al.*, 1995 ▸), it is possible to collect high-resolution patterns, without experimental noise, with sharp and well defined reflection profiles. These developments have facilitated the construction of high-resolution powder diffraction beamlines at several synchrotron sources (Hastings *et al.*, 1984 ▸; Cox, 1992 ▸; Fitch, 2004 ▸; Knapp *et al.*, 2004 ▸; Gozzo *et al.*, 2006 ▸). These powder experiments using diffraction filtering present very good signal-to-noise ratios and thin diffraction peaks having a mathematically well defined profile. This last point allows complex powder data to be collected and analysed quantitatively, notably by using Rietveld-type profile refinements – a method that was previously only allowed for data collected by neutron diffraction (Rietveld, 1969 ▸).

However, the selectivity of such a diffraction filter for powder diffraction experiments is strongly penalized by the low efficiency of the single-crystal analyser, as the ratio of the diffracted to incident intensities is low, especially when this analyser crystal is selective to improve the diffractogram angular resolution. This important disadvantage has led to the joint development of powder diffraction SR beamlines using either a 1D microstrip detector or a 2D detector (Bergamaschi *et al.*, 2010 ▸; Dyadkin *et al.*, 2016 ▸). However, this gain in measurement time does not compensate for the weakness of position-sensitive 1D or 2D detectors which usually do not allow very good angular and energy selection of the X-rays coming from the analysed sample. Their angular resolution is related to (i) the sample-to-detector distance, (ii) the size of the detector pixels and (iii) the size of the beam impact on the sample. Their background is increased by the air scattering of X-rays and by any enclosures around the sample (Bergamaschi *et al.*, 2010 ▸; Toby *et al.*, 2013 ▸). Furthermore, experiments using X-ray energies close to sample absorption thresholds are more difficult due to the presence of a high-fluorescence background, so for anomalous diffraction experiments it was also proposed to use energy-dispersive optics (simultaneous multiwavelength anomalous diffraction) that allow a differentiation of the paths of beams having different energies (Lee *et al.*, 1995 ▸; Lee & Ogata, 1995 ▸; Hodeau *et al.*, 1995 ▸).

Therefore, it is important to develop multi-filtering systems that are not penalized by the low efficiency of using only a single analyser crystal. For this reason, 25 years ago, developments combining several analysers/detectors were made using adjustable or fixed geometries (Toraya *et al.*, 1996 ▸; Hodeau *et al.*, 1996 ▸, 1998 ▸; Siddons *et al.*, 1998 ▸; Gozzo *et al.*, 2004 ▸; Fitch, 2004 ▸; Elkaïm *et al.*, 2007 ▸; Lee *et al.*, 2008 ▸; Thompson *et al.*, 2009 ▸; Peral *et al.*, 2011 ▸). In practice, the option of combining, generally less than ten, fixed analysers/detectors, arranged on a given profile, has proved to be the most easily achievable and reliable option. Indeed, even if its adjustment may not be entirely perfect, the imperfection of the data collected by each analyser/detector is essentially a simple and small shift in the value of the direction of its angle 2Θ_A_, a shift which can be constant for a fixed and stable diffraction filtering device, whatever the changes of objects to be analysed. So this imperfection can be mathematically corrected when summing the information given by the ten or so analysers/detectors, allowing excellent resolutions to be obtained (0.002–0.006°) and giving profiles described by a Gaussian and Lorentzian convolution function of the pseudo-Voigt-type which can be easily calculated (Hall *et al.*, 1977 ▸; Thompson *et al.*, 1987 ▸; Finger *et al.*, 1994 ▸). This advance allows quantitative analyses and also makes possible analyses of the intrinsic deformations and stresses of the samples.

As diffraction filtering has proven its efficiency, many of the high-resolution powder diffraction beamlines, which benefit from this and that have been built since the 1990s on various SR sources, are still in operation. This type of filtering has been achieved on these beamlines simply by the mechanical addition of several Θ/2Θ analyser + slit + detector systems in different ways: (i) addition of several analyser/detector arm assemblies (Toraya *et al.*, 1996 ▸), (ii) addition (fixed or adjustable) of several multi-analysers/detectors on the same Θ/2Θ arm (Hodeau *et al.*, 1998 ▸; Fitch, 2004 ▸; Knapp *et al.*, 2004 ▸; Gozzo *et al.*, 2004 ▸, 2006 ▸; Patterson *et al.*, 2005 ▸; Elkaïm *et al.*, 2007 ▸; Wang *et al.*, 2008 ▸; Lee *et al.*, 2008 ▸; Toraya, 2009 ▸; Peral *et al.*, 2011 ▸; Sitaud *et al.*, 2012 ▸; Llorens *et al.*, 2012 ▸, 2014 ▸; Fauth *et al.*, 2013 ▸; Bordessoule *et al.*, 2013 ▸; Dippel *et al.*, 2015 ▸; Sheu *et al.*, 2015 ▸, Schökel *et al.*, 2021 ▸) or (iii) both the above at the same time (Tartoni *et al.*, 2008 ▸; Thompson *et al.*, 2009 ▸). For these powder diffraction experiments, during the data collection the only variable is the rotation 2Θ of the detection arm supporting a multi-analysers by diffraction (MAD) system associated with several point detectors and, more recently on some beamlines, also associated with a 2D detector (Dejoie *et al.*, 2018 ▸; Fitch & Dejoie, 2021 ▸).

**Figure 1:**
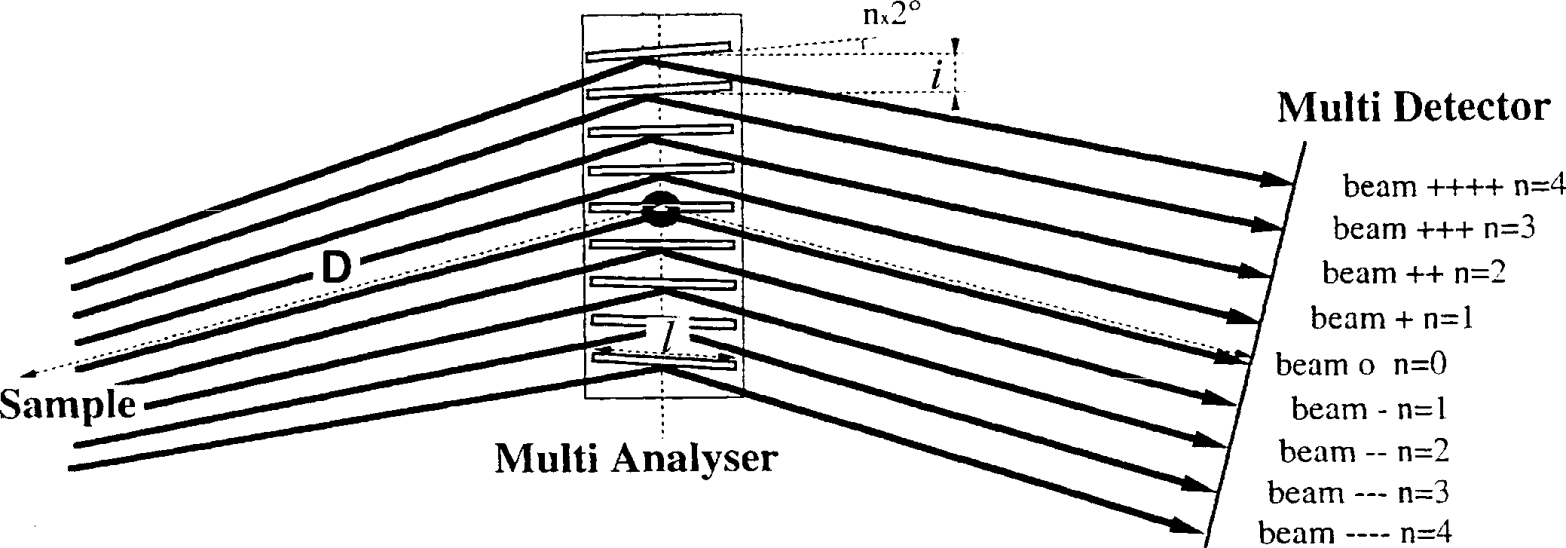
Scheme and beam paths on a multi-analyser diffraction (MAD) filter using nine analyser crystals with a 2° pitch between them; to avoid interception/cutting of the beams coming from the sample by the neighbouring analyser crystals, the maximum length of the analyser crystals *l* must be small and their spacing *i* must be relatively large; this is facilitated by the use of a large sample-to-MAD-filter distance *D* (from Hodeau *et al.*, 1998 ▸).

To increase the reliability of such MAD filters, it is important to design them with elements having a fixed and thus a stable geometry, where several analysers are associated in a compact and rigid way which facilitates their multiplication and allows several patterns to be collected in parallel. Such a multiplication allows a greater sensitivity to weak signals and partially compensates for the loss of intensity due to diffraction filtering. Each analyser/detector must be offset from the previous and the next ones by an angular pitch which, together with the length of each analyser, determines the MAD system size. In fact, this pitch has often been chosen close to 2°, a fairly large value which imposes a low number of analysers/detectors and therefore a restriction of the gain in diffracted intensity (Fig. 1 ▸). For example, if the offset between the axes of the analysers is close to 2°, such a system comprising nine analysers/detectors requires a scan of at least a 2° step, associated with a software correction of the measured efficiency of each channel (due to possible slight misalignments of the crystals and the efficiency of the detectors) and, if we want to ensure an overlap off all the channels, we have to use a rotation of the detection arm of more than 18°.

In such MAD multi-analyser/detector systems, the positioning and distance chosen between successive analysers/detectors also determines the usable energy range, as their design must ensure that each beam cannot be cut off by one of the adjacent analyser crystals (Fig. 1 ▸). Indeed, a variation in the energy of the X-ray source leads to a variation of the Bragg angle Θ_A_ and a significant variation in the paths of the diffraction filtered beams and their impact on the analysers. It is this constraint that has determined the choice, in most of the systems currently in use, of a relatively large distance between each analyser. This induces a large angular pitch/size of the analyser/detector system and requires relatively important minimum angular data collection to benefit from the full intensity gain of this system. It is therefore important to develop a MAD system, either continuous or with a smaller angular pitch/size, that allows an increase of the number of diffraction filtered channels and that allows a decrease in the minimum angular range for data collection. These constraints and the size and the distance *D* to the sample of the associated 1D–2D detector determine the usable energy range and the geometry of these MAD filtering systems.

## 3. Optimization of a compact-rigid diffraction multi-filtering system

**Figure 2:**
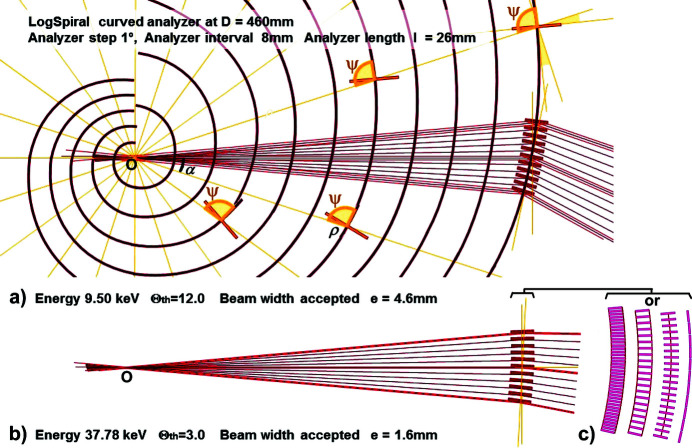
The positioning of several Si(111) analyser crystals on a logarithmic-spiral curve optimizes the paths of the beams coming from the centre *O* of this curve which impact with the same angle Ψ the middle of each analyser crystal; in this figure the crystals are tilted with a 1° pitch and the corresponding beam paths through this LogSpiral MAD filter are represented. (*a*) To avoid interception/cutting of beams coming from the sample, the maximum length of the crystals *l* and their spacing *i* are imposed by the lowest energy accepted by this rigid MAD system, here 9.5 keV, which corresponds to *l* = 26 mm and *i* = 8 mm for a sample-to-analyser distance *D* = 460 mm, and allow simultaneous measurements with an angular step of 1° and a beam width of 4.6 mm. (*b*) At higher energies, the same crystal size and spacing leads to a decrease in the width of the beams analysed to 1.6 mm at 37.78 keV. (*c*) This LogSpiral curved analyser surface can be realized either by a single thin curved crystal or by several crystals supported on a fixed and rigid dedicated curved surface.

The MAD multi-filtering devices studied here are essentially compact, rigid and fixed devices, as such systems allow high reliability and ease of use. With this option, the choice of geometrical parameters and the nature of the diffracting surface [Si(111), Ge(111), C(002),…] are the constraints that will define the X-ray spectral range of their operation, their experimental range of use and their possibilities of applications (SR source and, possibly, laboratory X-ray tube source). The devices that we propose *in fine*, are based on the realization of a dedicated analyser crystal having diffracting planes curved along directions such that each part of its surface allows the diffraction filtering of a well defined incoming beam. In order to optimize the impact of the incident beam on the middle of each analysing crystal, this MAD filter uses a filtering surface having, for one of the two directions of its curved surface, a logarithmic spiral (‘LogSpiral’) mathematical function (Sakayanagi, 1982 ▸; Wittry *et al.*, 1993 ▸; Zhong *et al.*, 1999 ▸; Toraya, 2009 ▸). The characteristics of this curve mean that any point on it always sees its origin *O* under the same angle Ψ; its radius of curvature is *R* = ρ/sin(Ψ) and its polar equation is ρ = aexp[αcot(Ψ)] (Fig. 2 ▸). The MAD filter has this curvature over a small length and its crystal planes will be the diffraction planes of the analyser; so its curvature is such that the angle Ψ corresponds to the Bragg angle Θ_A_ which is determined by the value of the X-ray energy used for the experiment and the *d*_*hkl*_ spacing of the diffracting planes of the analyser. This curved surface can be either a crystal, a thin film or multiple crystals supported on a fixed and rigid dedicated curved surface [Fig. 2 ▸(*c*)]. Figs. 2 ▸(*a*) and 2(*b*) show, for energies close to 9.5 and 40 keV, the beam paths and beam widths of a diffraction filtering system made with several Si(111) single crystals, positioned on a LogSpiral curve with a step of 1°, which operate at a sample/MAD distance *D* = 460 mm. The width of the filtered beam changes as the source energy changes and, at low energies below 9.5 keV, the experiment is impossible because the filtered beam paths are partially intersected by the adjacent analyser crystals. It is therefore important to calculate and optimize several geometry options to perform such diffraction filtration over the widest experimental analysis angular range, applicable over a respectable X-ray energy range. In the different set-ups designed and tested, the LogSpiral curve was used along one of the two directions of the surface, and for the other direction the curve was either a small straight line or a portion of a circle.

The concrete realization of such a MAD system requires the creation of a rigid surface with a very precise dedicated curvature which is constant over time and can be costly to achieve. However, with the new means of 3D printing, it is now possible to produce supports having complex geometries on which we can fix such a MAD diffraction filter (thin single crystal, thin layer or single-crystal array). For each analysis geometry, we need to optimize the configuration of the continuous or individual diffracting elements and their assembly mode (precise, fixed and compact) that allow the required efficiency for filtering in the desired X-ray energy range. If we use a 3D printing process, this MAD system can be realized at a reasonable cost but it is necessary to produce a rigid system which requires a design that avoids stresses on the diffracting surfaces. This compact-rigid MAD multi-analyser system should also allow the combination of Soller-type or other collimator assemblies with the need of an optimal and rigid alignment. All these points determine the final choice of characteristics, geometries and modes of association of the diffracting elements on this LogSpiral curved surface, their positioning precision and their costs. The gain in sensitivity brought by such a development may allow the fields of application of such MAD multi-filters associated with 1D–2D detectors to be widened and to go beyond the restricted field of SR sources to that of laboratory diffractometers (for example with an Ag *K*α_1_ type X-ray source associated with parallel optics). In order to optimize such compact multi-filtering analysis devices, we realized and tested several MAD multi-filtering configurations with a curved surface of diffracting planes using different diffraction geometries: (*a*) continuous reflection (§3.1), (*b*) transmission (§3.2) and (*c*) discontinuous reflections (§3.3).

### 3.1. Compact MAD multi-analyser system with a curved support surface parallel or slightly tilted to the crystal diffraction planes, operating in reflection mode – (*a*)

**Figure 3:**
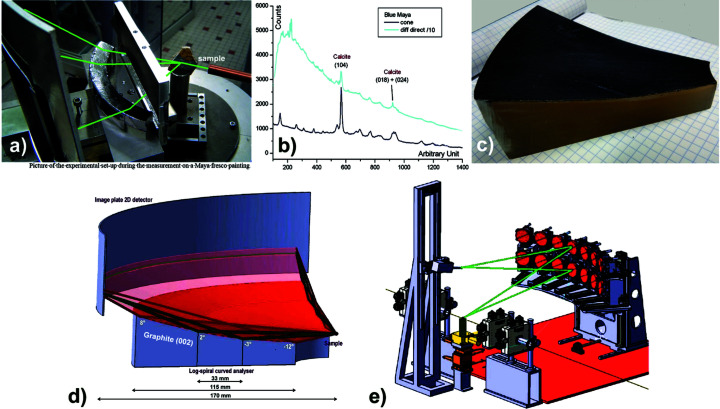
Examples of bi-curved crystal analysers having a support parallel or slightly inclined to the diffraction planes and operating in reflection mode. (*a*) Photograph of such a system having a ‘plan + circular’ profile of graphite (002) planes and tested with a Cu *K*α_1_ X-ray source; beam paths are in green (Hodeau *et al.*, 2008 ▸). (*b*) Example of a diffraction pattern collected by this system on a Maya fresco painting, without (blue) and with (black) diffraction filtering: the diffuse background filtered by the cone is ∼30 times weaker than the unfiltered one, while the amplitude of the (104) diffracted peak of the calcite filtered by this graphite (002) analyser is only about three times weaker than the unfiltered one; thus with this diffraction filtering, the signal-to-noise ratio is much better and the fluorescence is suppressed, allowing clean diffraction patterns of this heterogeneous sample to be collected. (*c*) Realization of the MAD analyser in reflection mode, having a double ‘logarithmic-spiral + circular’ profile, by using a 3D printed LogSpiral support with a 0.5 mm graphite (002) sheet glued on this curved surface. (*d*) 3D scheme of such a MAD system having a ‘LogSpiral + circular’ profile: this figure shows beam paths on this bi-curved analyser having a crystalline graphite mosaic layer (002) optimized for filtering Cu *K*α_1_ X-ray radiation. In this example the sample-to-detector distance is 170 mm and the radial width used on the MAD system is chosen to be 33 mm or 115 mm: this increase in the usable dimension of such a system increases its angular filtering acceptance from 5° (dark green lines) to 20° (dark brown lines) and the width of the readout band on the 2D detector (from 1 to 15 mm); it should be noted that this band is very thin (<1 mm) for an angular acceptance of 5° (Hodeau *et al.*, 2008 ▸). (*e*) Example of the 14-crystal Si(111) spherical plate spectrometer (having 500 µm thin sheets of glued single crystal) developed on the ESRF FAME-UHD line; this spectrometer is dedicated to accurate fluorescence detection; the beams filtered by each analyser (as seen by green lines for one diffraction plane) have a rather large width which is related to the size/geometry of its support dish and allows the filtering efficiency to be increased (Hazemann *et al.*, 2009 ▸; Llorens *et al.*, 2012 ▸; Proux *et al.*, 2017 ▸).

This geometry allows the inclusion of a diffraction filter on a diffraction instrument which can be used with a 1D–2D detector over a wide angular range for powder diffraction and which allows the collection of its complete diffraction pattern without including an overly complex system on the instrument. Such an analyser surface has been tested with a simple bi-curved conical analyser (whose two directions are a circle and a straight line) using the 002 reflection of a graphite crystal as diffraction filter. The realization of this system was performed by using a deposit of pyrolytic graphite on this surface and its efficiency was reported in patent DI011169-01 and demonstrates the principle of the system (Hodeau *et al.*, 2008 ▸). In the experimental set-up shown in Fig. 3 ▸(*a*), the profile is straight in the diffraction plane and the filtered intensity obtained is quite low (with a decrease by a factor of one-third) even if we use a graphite analyser with a wide bandwidth (0.3–0.5°) (Gianoncelli *et al.*, 2008 ▸). Nevertheless, such an analyser provides a good gain in signal-to-noise ratio; furthermore, it allows all fluorescence to be suppressed [Fig. 3 ▸(*b*)]. As its angular dimension in the second direction, along the circumference of the MAD filter, can be large (from 10° to 150°), such a system can allow fast and continuous measurements over the whole diffractogram. This study was carried out to work with a Cu *K*α_1_ X-ray energy, so this MAD filter is easy to use and has been tested with a conventional laboratory X-ray tube source. Such systems can be developed and are of interest for portable diffraction equipment (Gianoncelli *et al.*, 2008 ▸; Chiari *et al.*, 2008 ▸; Nakai & Abe, 2012 ▸; Beck *et al.*, 2014 ▸; Castaing *et al.*, 2016 ▸).

To increase the efficiency of the diffraction filtering by such a system, it is possible to widen the angular range accessible by the beam in the other direction: replacing the straight profile in the radial direction of the analyser plate by a logarithmic-spiral profile [along the radial/horizontal direction in Fig. 3 ▸(*d*)] can increase the angular acceptance in the diffraction plane of such a curved MAD analyser from 0.5° to 20°; its efficiency can therefore be increased by a factor of 20/0.5 = 40. The gain in intensity of using this MAD analyser associated with a 2D detector would then be significant with a conservation of its gain in signal-to-noise ratio. The realization of a crystal having such a bi-curved shape can be difficult and its cost can be quite important; however, with 3D printers it is possible to produce, at a modest cost, support having a bi-curved surface: LogSpiral in one direction and a portion of a circle in the other direction. Fig. 3 ▸(*c*) shows an example of such a (002) graphite MAD system, made with a geometry that allows experiments in reflection mode using a Cu *K*α_1_ energy X-ray source. It is made with a mosaic crystalline graphite sheet parallel to the (002) planes, available in a 0.5 mm thickness, which is deposited and glued onto a 3D printed bi-curved polymer support. Fig. 3 ▸(*d*) shows two examples of beam paths filtered by such a bi-curved MAD filter located at a sample-to-detector distance *D* = 170 mm: (i) if its used radial dimension is less than 33 mm, its vertical angular acceptance is 5° and the width of the reading line on the 2D detector is 1 mm (dark green beam paths), and (ii) with the same sample-to-detector distance and with a larger used radial dimension (115 mm) of the analyser system, its angular acceptance becomes close to 20° and the width of the signal received on the detector is a band about 15 mm wide (dark brown beam paths) and the intensity is proportionally greater.

On such a bi-curved support, it is also possible to replace the mosaic crystalline sheet of graphite (002) by a pavement of small perfect Si(111) crystals diffracting in reflection mode, as has already been done for spectroscopy analysers such as that of the FAME-UHD beamline of ESRF (Hazemann *et al.*, 2009 ▸; Llorens *et al.*, 2012 ▸; Proux *et al.*, 2017 ▸), which is dedicated to the precise detection of the fluorescence of diluted materials or for highly resolved measurements of absorption threshold structures [Fig. 3 ▸(*e*)]. With such a configuration the resolution and filtering are much better and this system allows ultrahigh-dilution X-ray absorption spectroscopy measurements for trace-element studies (especially sub-p.p.m. concentrations) in samples of environmental, chemical or biological interest. For such a bi-curved analyser, one can also use a curved quartz crystal cut according to a network of (244) planes and slits, diffracting in reflection mode, as was used for the RIXS spectrometer on the P01 beamline of PETRA III at DESY (Ketenoglu *et al.*, 2015 ▸).

### 3.2. Compact multi-analyser system with a curved support surface perpendicular or strongly inclined to the diffraction planes, operating in transmission mode (Laue) – (*b*)

**Figure 4:**
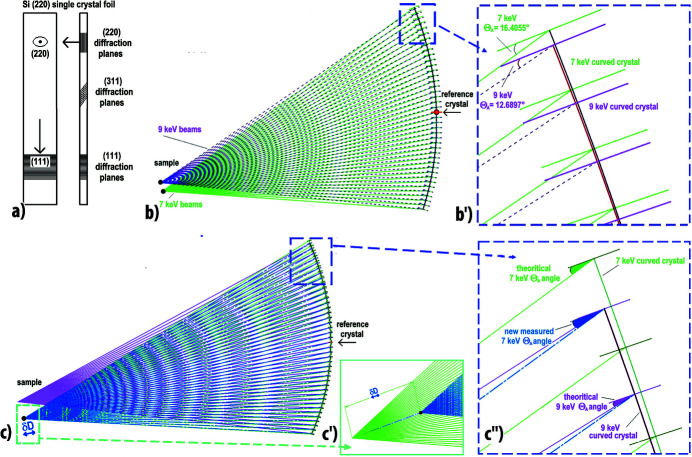
Optimization of the curvature of crystal sheets to realize Laue mode analysers; along its curvature, at every 1°, the diffraction planes of these thin curved crystals are symbolized by a large plane with the corresponding direction of its incident X-ray beam. (*a*) Examples of crystal plane orientations for positioning the thin Si single-crystal sheet in a transmission MAD system. (*b*) Beam paths from two sources at 7 keV and 9 keV (green and purple, respectively) arriving on the Si(111) planes of two single-crystal sheets which have ideal curvatures for 7 keV and 9 keV, respectively; the origins of these representations have been translated so that the midpoints of the two curved crystal sheets are coincident. (*b*′) Zoom-in on the directions and impacts of the beams at the upper ends of the single-crystal sheet which enlarge the most important curvature difference existing between 7 keV and 9 keV (green and purple), an energy range close to that of a Cu *K*α_1_ X-ray source. (*c*) Beam paths coming from these two different energy sources 7 keV and 9 keV, positioned to be filtered by Si(111) planes of a single-crystal sheet having its curvature determined for 9 keV in both cases; the purple curve represents the ideal paths of the 9 keV beams filtered by a MAD system having an ideal curvature for 9 keV, the blue curve represents the paths of the 7 keV beams filtered by a MAD system having a modified curvature corresponding to 9 keV filtering and corrected due to a δ*D* optimization of the sample-to-MAD-system distance. (*c*′) Enlargement showing this variation δ*D* in the distance of the sample from the MAD system which can modify and reduce the deviations of the Bragg angle Θ_A_ of the beams on this curved crystal of the MAD filter and can allow a wider angular range of filtering, as discussed in the text and in Fig. 5 ▸. (*c*′′) Zoom-in on the impact of these 7 and 9 keV beams, at the upper ends of the single-crystal sheet with curvatures determined for 9 keV ideally and with the modification due to a small δ*D* variation of the sample-to-MAD-system distance.

Such MAD filters can also be made using a large thin crystal which allows it to be bent and used for diffraction filtering in Laue transmission mode. Depending on the crystallographic orientations and the asymmetric cuts of this thin single crystal sheet, we can select the diffraction planes used for filtering. The thickness of the crystal must be relatively small to reduce its stress and absorption and ultimately to optimize the filtered intensity and the resolution of the transmitted beam, as was done for Laue monochromators (Lee *et al.*, 1994 ▸; Hagelstein *et al.*, 1995 ▸). The transmission of the crystal must be sufficient (of the order of 30–50%) to allow good reflectivity of the diffraction reflection used for filtering, but the crystal sheet must also be thin enough to allow its curvature while maintaining low stresses and a small reflection width (*e.g.* ∼0.5 eV). Depending on the energy range of use, this dictates the nature of the analyser crystal and its thickness (*e.g.* ∼200–100 µm of silicon). The crystal orientation of this foil is guided by good reflectivity, *e.g.* the size/orientation of a silicon crystal can be chosen to have the (111), (220) or (311) planes perpendicular to the curved foil [Fig. 4 ▸(*a*)]. Although the [111] direction is the best for filtering with good intensity, we can also use the [311] direction to have a larger diffraction angle Θ_A_ with X-rays corresponding to the Mo *K*α_1_ emission energy.

If the curvature of this single-crystal foil follows a logarithmic spiral shape, its response and efficiency will be optimal for making a transmission MAD filter. If this Laue-mode analyser sheet is far away from the sample to analyse, its radius of curvature is relatively large, allowing moderate strain and a good resolution filtering. A MAD filter having this configuration was developed in the late 1990s on the ID BioCAT beamline at the Advance Photon Source for fluorescence XAFS experiments on dilute samples (Zhong *et al.*, 1999 ▸) and the crystal of their analysing filter, which diffracts in the Laue mode, is curved in a logarithmic spiral shape. The system developed on this APS BioCAT ID beamline uses a thin Si(111) analyser crystal with an asymmetric cut (thickness 200 µm) curved with an average radius of ∼200 mm; it allows filtering, with a reflectivity of ∼10% for fluorescence measurements at the Ag *K* edge. Such MAD filtering using a curved crystal in Laue geometry is efficient and was later developed on several spectroscopy beamlines (Zhong *et al.*, 1999 ▸; Kropf *et al.*, 2003 ▸; Barrea *et al.*, 2010 ▸; Wu *et al.*, 2013 ▸; Wakisaka *et al.*, 2020 ▸). As the curvature parameters of the logarithmic spiral curve depend on the diffraction angle Θ_A_ of the analysing filter, several systems have to be made for different X-ray sources or for XAFS experiments performed at different emission/absorption energies. As an example, about ten systems have been proposed and are marketed for use at energy ranging from 5 keV to 22 keV (Oxford Danfysik BCLA, https://fmb-oxford.com). In the following, we present some parameters and geometrical adjustments for the positioning of such a MAD filter in Laue transmission mode, in order to extend its range of use. In particular, we have studied the curvature deviations associated with variations of X-ray energies to be filtered, for the three energy bandwidths 7–9 keV, 10–20 keV or 20–30 keV, close to laboratory sources of the Cu *K*α_1_, Mo *K*α_1_ or Ag *K*α_1_ type.

To evaluate the conditions of use of such a system over an energy range (*e.g.* 7–9 keV), two sets of beam paths (green and purple) corresponding to the limiting energies of this range are superimposed in Fig. 4 ▸ to show the differences in the beam paths but also the extreme differences from the ideal curvature of the Si(111) analyser crystal, taking the midpoint of the central analyser crystal and the tangent of the curvature at this point as a common reference. From the superposition of the two extreme curvatures of this analyser crystal sheet we can see in Fig. 4 ▸(*b*) that the two sources/sample positions (corresponding to the ideal position of the sample in relation to the analyser curvature) are not the same: there is a vertical shift due to the variation of the Bragg angle Θ_A_. It can also be seen that the difference in curvature between the two energies (7 and 9 keV) is quite small, with an angular variation of the angle of impact of the beam (and therefore of its deviation from the ideal Bragg angle Θ_A_) that increases as one moves away from the central crystal, *i.e.* as the angle range of use of the curved crystal increases (*i.e.* its working length). Fig. 4 ▸(*b*′) enlarges the beam paths by focusing on the upper ends of the curved analyser which has the largest deviations in curvature and in angle, and highlights the main differences in these two curvatures/beam-paths/angle Θ_A_.

To better simulate and quantify effects of MAD curvature changes, we superimpose in Fig. 4 ▸(*c*) a single Si(111) sheet with the curvature optimized for 9 keV with the ideal paths of beams from a 9 keV energy source (pink), but also a modified curvature (from green to blue beam paths) corresponding to 9 keV filtering but corrected to receive 7 keV beams using a δ*D* optimization of the distance between the sample and the MAD system resulting in a small horizontal shift of the sample position [Fig. 4 ▸(*c*′)]. We can visualize the small difference between the theoretical Bragg angles Θ_A_ for 7 keV beams and those used by intercepting a MAD analyser curve calculated for 9 keV with these δ*D* optimizations; this difference is also more significant at the upper ends of the curved analyser [Fig. 4 ▸(*c*′′)]. Since diffraction filtering with such a curved and thin analyser filter is only possible if the agreement between the observed Θ_A_ angles and the theoretical Bragg Θ_A_ angles is better than ±0.03° (Zhong *et al.*, 1999 ▸), the angular range of use of such a curved analyser can be very small (a few degrees). It is therefore important to optimize and extend the range of use of the MAD filter system by minimizing this angular deviation from the angle existing with an ideal curvature, by using a small variation δ*D* in its distance from the source.

**Figure 5:**
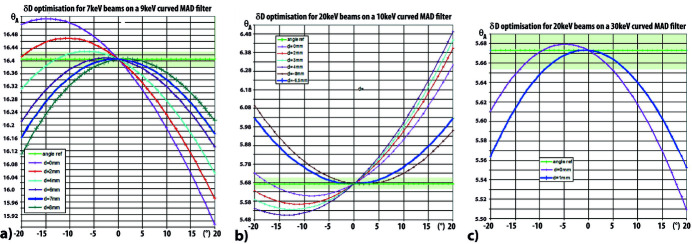
Optimization of the distance *D* − δ*D* between the source/sample and the Laue mode curved MAD system; for some small δ*D* displacements, representations of the real values (thin colour curves) of the Bragg angles Θ_A_, compared with the ideal values (green lines) (calculated for a beam of 7, 20 then 20 keV, arriving on an analyser having a curvature optimized for 9, 10 then 30 keV, respectively). (*a*) Variation of Bragg angles Θ_A_ according to different *D* − δ*D* distances for a 7 keV beam arriving on an analyser having a curvature optimized for 9 keV. (*b*) Variation of Bragg angles Θ_A_ for a 20 keV beam arriving on an analyser with a curvature optimized for 10 keV. (*c*) Variation of Bragg angles Θ_A_ for a 20 keV beam arriving on an analyser with a curvature optimized for 30 keV. For these three energy ranges (7–9 keV, 10–20 keV and 20–30 keV), close to Cu *K*α_1_, Mo *K*α_1_ and Ag *K*α_1_ laboratory X-ray sources, a small displacement δ*D* (7 mm, −6.5 mm and 1 mm, respectively) widens the accessible angular range of filtering (∼20°, ∼15° or ∼20°, respectively) (dark blue curves).

This optimal variation δ*D* of the distance *D* between the sample and the curved MAD system can be calculated for different energy ranges. Figs. 5 ▸(*a*), 5(*b*) and 5(*c*) report, for several values of this distance *D* − δ*D*, the variations of the deviations of the Bragg angles Θ_A_ from its ideal value (green line) in the three operating ranges 7–9 keV, 10–20 keV and 20–30 keV. In these figures the green line and the light green band are the ideal and acceptable value of the Bragg angle Θ_A_, respectively, and the dark blue curve corresponds to deviations calculated for the optimal δ*D* shift giving the widest angular range of diffraction allowed for these transmission MAD filters. We see that, for these three energy ranges, a small δ*D* shift can increase the optimal angular range of the accessible experimental measurements by a factor of 2 to 5 and this gain depends on the values of the reference energy. It is easy to achieve/optimize this adjustment by such a simple δ*D* shift of the distance of the whole MAD system relatively to the sample/source and this process can also facilitate the optimization of the angular range of use of this type of MAD filtering for experiments using laboratory X-ray sources.

**Figure 6:**
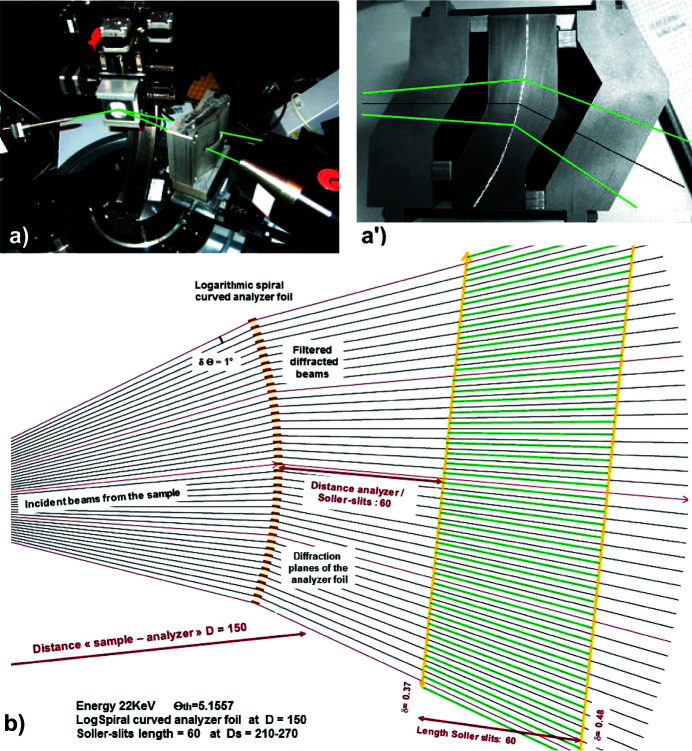
Compact MAD analyser block with a thin LogSpiral curved analyser crystal perpendicular to its Si(111) diffraction planes and working at a distance *D* = 150 mm in Laue transmission mode with X-rays having the Cu *K*α_1_ energy. (*a*) Photograph of this setup during a laboratory experiment (beam paths are in green). (*a*′) Enlarged photograph of such a compact MAD block working in Laue transmission mode (beam paths are in green). (*b*) Schematic representation of beam paths filtered by this curved MAD filter and then cleaned/separated by a Soller collimator having long blades (60 mm) located and optimized for the distance *D*_s_ = 210–270 mm.

For the construction of such a thin and curved single-crystal analyser it is important to manufacture sandwich-type support blocks that stiffen its LogSpiral curvature [Figs. 6 ▸(*a*) and 6(*a*′)]. In a first prototype calculated, built and tested to operate at a distance of 150 mm from the sample, we used Si(111) crystal planes to act as the analyser filter. The calculations carried out with this configuration gave a reasonable resolution (∼1 eV), wider than the intrinsic width of a perfect Si(111) crystal, due to the low thickness of the analyser sheet and the stresses related to its curvature. On the other hand, these test measurements highlighted a poor signal-to-noise ratio of this first configuration in transmission mode. Indeed, all the scattering, linked to the experimental setup, passes partly through the thin single-crystal sheet of silicon. We modified this MAD system with one or two (rather archaic) configurations of short pre- and post-analyser slits which did not, however, give sufficient signal-to-noise gain. To solve this problem, we propose to replace a limited number of short slits (10 mm) by a long and dense Soller-type collimator (60 mm) [Fig. 6 ▸(*b*)], which will greatly reduce the angular acceptance.

It is important that this Soller collimator is at a relatively large distance ‘*D*_s_’ from the sample to be analysed, so that each slit corresponds to an angular filter as fine as possible. As an example, if the distance *D*_s_ of the Soller collimator to the sample is increased from 210 mm to 960 mm, we can isolate beams that are angularly separated by 1–0.5° to 0.1°, respectively. However their insertion between the curved analyser crystal and the 2D detector implies that the diffraction filtering is no longer done in continuous mode over the whole angular range of measurement because there are shading zones related to the presence of each blade of the Soller collimator: during the experiments it is therefore necessary to scan at least the angular range existing between these blades/slits (1–0.5° or 0.1° for the distances *D*_s_ = 210 mm or 960 mm, respectively).

### 3.3. Compact multi-analyser system with a curved support surface perpendicular or strongly inclined to the diffraction planes, operating in reflection mode – (*c*)

In order to use such a MAD system in reflection mode, the curved surface must be discontinuous to allow the beams to pass through. We have studied two types of compact ‘MAD blocks’ having a logarithmic spiral curvature and allowing the realization of a series of analyser crystals:

(c1′) – (§3.3.2) A compact-rigid multi-crystal analyser block formed by a ‘sandwich-comb’ of Si(111) crystals which allows a sequence of analyser crystals with good alignment precision and which can be realized with a step of 0.2° between perfect single crystals. It can be made with individual silicon single crystals placed in a holder having 3D printed slots so that all centres of the crystals are located on a LogSpiral curve with a 0.2° inclination between each Si(111) crystal plane. Easy to construct and inexpensive, it must be associated with an appropriate ‘Soller-collimator’ block. Its geometry can be optimized for use over a given energy range of the X-ray source, but in this case its angle with the Soller-collimator varies with the Bragg angle Θ_A_.

(c1′′) – (§3.3.3) A single compact block containing both ‘sandwich-comb + Soller-collimator’, where the multi-crystal analyser and the Soller multi-blades are rigidly associated. This choice of stiffening these two systems imposes that such a MAD system must be designed to operate with a single energy source. Indeed, its shape being rigid, the angle Θ_A_ between the block of single crystals and the block of collimators must be constant to allow such a common support. It is preferable to design it for high energy (*i.e.* for an Ag *K*α_1_ source) as this make it easier to optimize the beam paths of each single crystal; such a system can be achieved at low cost.

(c2) – (§3.3.4) A compact-rigid-curved MAD filter realized from a ‘single-crystal-comb’ generating Si(111) planes which are curved by the confinement of this comb on a rigid support having a LogSpiral curvature. This realization allows a high density of identical analyser crystals with high alignment accuracy allowing a step of 0.1° between these perfect single crystals. The design of such a block and the size of the comb were studied in order not to generate stresses in the Si(111) crystal blades during its stiffening. Its construction is more complex but can be achieved at a reasonable cost; it must also be combined with a Soller-collimator block.

For these configurations c1′, c1′′ and c2, the curved surface of the support must be discontinuous to allow the beams to pass through the support. The total angular dimensions of this curved surface can be quite large (2°–5°–10° or more), to be adapted to the size of the 1D–2D detector. We can built a comb containing 20–50–100 analyser crystals tilted successively by 0.1° or 0.2°. Such a density/geometry would allow much faster measurements (equivalent to 20–50–100 parallel data collections), but it still requires a small partial scan of the 2Θ arm which carries the full MAD system (MAD filter + Soller-collimator + 1D–2D detector). To test the geometry of these c1 and c2 MAD blocks, we have chosen Si(111) crystals as diffracting crystals because their resolution is very good and their cost is reasonable. The high-energy limit of such a system is mainly related to the length of the Si(111) crystals forming the comb. The low-energy limit of use is related to the gap between adjacent Si(111) crystals which also imposes a large distance *D* between the sample to be studied and the MAD block. In the configuration we tested, the parameters of the logarithmic spiral curve were determined using a large distance *D* = 973 mm to allow a small angular pitch (0.1°) between the Si(111) crystals with a reasonable distance between the crystal blades (1.7 mm). These parameters allow a high density of crystals in a small volume as well as a wide range of usable energy (from 22 keV to 46 keV). The choice of this energy range is important because it allows these systems to work with a high-energy SR source (which is the case for the majority of powder diffraction beamlines today), but also to be able to work at the Ag *K*α_1_ energy which allows its use in the laboratory with a tube-type source associated with a parallel optic.

#### 3.3.1. Sandwich-comb analysers – (c1)

**Figure 7:**
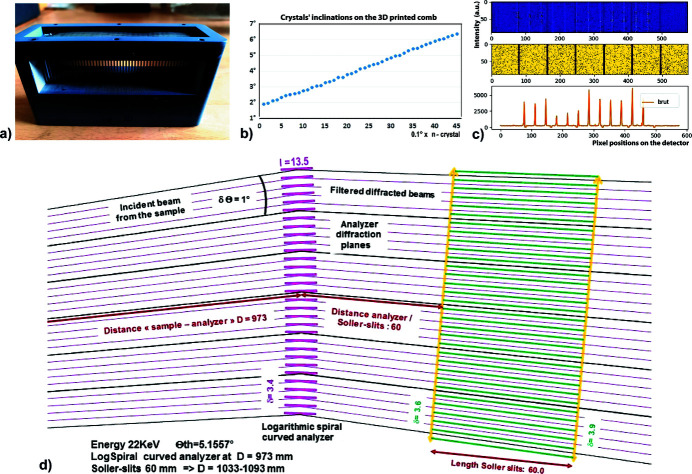
Sandwich-comb analyser block with Si(111) crystals working in reflection mode. (*a*) Sandwich-comb made by 3D printing. In this example we inserted a Si(111) single-crystal plate into all of the slots spaced 1.7 mm apart and corresponding to a crystal pitch of 0.1°. (*b*) Angular measurements of the beam filtered by this block and therefore of the tilt of the analyzer crystals. (*c*) Diffraction measurements filtered by a sandwich-comb having an angular pitch of 0.2° between the Si(111) crystals and being associated with a Soller-collimator block having a pitch of 0.1° (due to the unavailability during this experiment of a Soller collimator with a 0.2° pitch), so this mismatch led to more restrictive intensity cuts of the filtered beams; this Soller-collimator block is fixed on the 2Θ_A_ arm at a distance (*D*_s_ = 1033–1093 mm) from the sample in front of the hybrid photon-counting XPAD S70 detector (Basolo *et al.*, 2007 ▸; Medjoubi *et al.*, 2010 ▸); this 2D detector is made up of a succession of seven chips supporting pixels (yellow zones) and the dimension of each pixel of the XPAD S70 detector is 125 µm. (*d*) Scheme of 22 keV beam paths filtered every 0.2°, over a range of 6°, by two rigid blocks: the sandwich-comb of Si(111) crystals and the Soller-collimator. The distance between the sample and the MAD block is large (*D* = 973 mm).

A sandwich-comb of Si(111) crystals can be made by 3D printing to associate several dozen Si(111) crystal wafers on a rigid support having slots. These individual single crystals are placed in the slots of a 3D polymer holder so that the crystals follow a LogSpiral curve with a fixed tilt of 0.1° between each crystal. Our 3D printed holder was made using a multi-jet printing apparatus – ProJet MJP 3600. On the prototype that we made, the angle between each crystal was initially chosen to be very small (0.1°) in order to test the case of a very compact system with a very high density of crystals placed at a large distance *D* = 973 mm from the sample. To build such a block, we printed slots spaced 1.7 mm apart with an angular variation of 0.1° between them [Fig. 7 ▸(*a*)]. To test the accuracies of the alignments, we have inserted in this comb block Si(111) wafers every one, two or three slots corresponding to a crystal pitch of 0.1°, 0.2° or 0.3°.The size of the Si(111) single crystals is 30 mm × 13.5 mm × 0.8 mm finalized by low-stress optical polishing (Δ*E* ≃ 0.5 eV). Measurements of their angular orientations, carried out with 30 keV X-rays on the ESRF FAME beamline (Proux *et al.*, 2005 ▸), show that in such a comb the precision of the Si(111) crystal plane alignments could be relatively good [Fig. 7 ▸(*b*)]. This angular accuracy is mainly related to the good agreement between the width of the slots of the support and the thickness of the Si(111) blades. The best agreement is obtained when the width of the support slots is just slightly smaller than the thickness of the Si(111) wafers without the addition of glue; this is so that their small orientation deviations do not vary over time; however, this adjustment should not be too tight so as not to generate stresses in the Si(111) single-crystal wafers. Using this process, without additional mechanical adjustment, we can achieve an accuracy of the order of 0.05°–0.1° [Figs. 7 ▸(*a*) and 7(*b*)], which allows us to produce a comb of perfect single crystals with a pitch of 0.2°.

#### 3.3.2. Sandwich-comb analysers for use over a range of energies – (c1′)

This sandwich-comb of Si(111) crystals is mounted on the Θ_A_ axis of the analyser arm of the diffractometer, which allows it to adjust its angle Θ_A_ according to the energy of the experiment. This comb is associated with a Soller-collimator block at a distance *D*_s_ = 1033–1093 mm to the sample, also made by 3D printing, which allows the separation of the filtered beams by long (30 mm × 60 mm × 0.7 mm) thin absorbent blades (stainless steel or tungsten) spaced from 3.6 to 3.9 mm. This Soller-collimator block is mounted on the 2Θ_A_ arm in front of the 2D detector, by means of two vertical/horizontal translations and a goniometer head, in order to adjust its positioning/orientations to associate each beam path with a slot. Fig. 7 ▸(*d*) shows an example of beam paths filtered every 0.2° by these two rigid blocks. It is easier to have a large distance between the sample and the MAD block (here *D* = 973 mm) so that the small angular pitch (0.2°) between the Si(111) crystals corresponds to a sizeable distance between them (δ = 3.4 mm).

We tested this sandwich-comb analyser associated with a Soller-collimator block on the D2AM line at the ESRF (Ferrer *et al.*, 1998 ▸; Basolo *et al.*, 2007 ▸; Chahine *et al.*, 2021 ▸) by measuring the diffraction reflections of a reference LaB_6_ powder, obtained with a quasi-parallel beam of 0.4 mrad at an energy of 22 keV close to that of a laboratory Ag *K*α_1_ source and using a large distance *D* = 973 mm between the sample and the sandwich-comb block. These experiments show a very good reproducibility of the response of this ‘comb’ for a given position/fixation mode. They also show a sensitivity of the filtering system response to the match between a possible residual deviation δΘ_A_ of the crystal tilt angle from the ideal angle 2Θ_A_ and the adjustment of the filtered beam to the Soller-collimator position: our measurements indicate that small angular variations of such a sandwich-comb are too large to allow good beam separation with an angular pitch of 0.1°. However, this sensitivity of the crystal responses to the alignment of the system geometry can easily be relaxed by increasing the angular pitch between adjacent analyser crystals, as shown in Fig. 7 ▸(*c*); this sandwich-comb allows diffraction filtering using an angular pitch of 0.2° between the Si(111) crystals. For data collection, a single scan of the 2Θ arm integrates the different beams filtered by each crystals.

This compact-rigid sandwich-comb of Si(111) crystals, combined with a Soller-collimator block, is easy to build and has a low cost. This system allows a good separation of the signals filtered by perfect crystals with a pitch of 0.2° which can be used in parallel with a reasonable alignment precision. Such a MAD system can be used over a wide energy range (22 keV to 46 keV), which allows it to be used on a SR source, but also in the laboratory for experiments using an Ag *K*α_1_ source with parallel optics. In this case, it can be adapted by using mosaic or stressed crystals in order to increase their reflectivity (but with a loss in resolution).

#### 3.3.3. Double-sandwich-comb analysers + Soller-collimator for single energy use – (c1′′)

The alignment between the paths defined by the orientations of the Si(111) crystals and the channels of the Soller-collimator is fundamental: to avoid a misalignment between them with time, we can realize by 3D printing a support containing a double lattice of slots that determines and fixes the relative positions of the Si(111) single crystals and the collimator blades. This can be achieved by combining 25–50 Si(111) crystal blades with the collimator blades at a pitch of 0.2° on a single rigid support. However, as the angle Θ_A_ between the analyser crystals and the collimators must vary according to the energy of the X-ray source, such a mono-block system can only be designed for single-energy use and requires good stiffening of the support between the MAD system and the Soller-collimator. In this case, the only possible adjustments are the value of the diffraction angle of the multi-analysers+collimators block, its alignment parallel to the diffraction plane and its position/distance *D* from the sample (*i.e.* the centre of the diffractometer). The advantage of this single-block system is a good fixed separation of the different beam paths, but this comes at the cost of greater precision in the manufacture of its single double-sandwich-comb block containing both MAD-filter and Soller-collimator, and to adjust them correctly during its construction to obtain rigid channels with fixed beam paths separated every 0.2°.

#### 3.3.4. Single-crystal-comb analysers for use over a range of energies – (c2)

**Figure 8:**
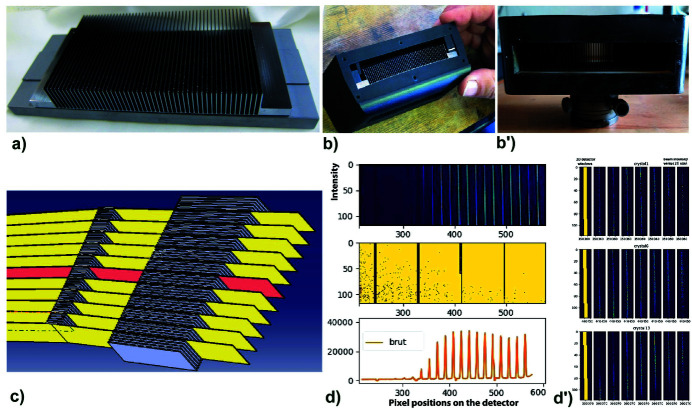
Single-crystal-comb MAD block having 50 Si(111) crystals working in reflection mode to filter powder diffraction patterns. (*a*) This silicon single-crystal-comb was cut to generate Si(111) planes 1.7 mm apart. Its single-crystal framework is perforated underneath and has a small thickness at the crystal junction (0.7 mm) to allow a few degrees of bending without generating stresses inside the Si(111) crystal blades. (*b*) Photograph of the single-crystal-comb block made by 3D printing that imposes the LogSpiral curvature to the silicon single-crystal-comb; it is mounted on the Θ_A_ arm of the diffractometer. (*b*′) Photograph of the 3D printed Soller-collimator block containing 51 collimator blades. It is mounted on the 2Θ_A_ arm of the diffractometer after the single-crystal-comb block and before the 2D detector. (*c*) Scheme with 46 keV X-rays (Θ_A_ = 2.4632°) of 11 paths of the 50 beams (spaced by 0.1°) and filtered by this rigid compact analyser block associated with the Soller-collimator block. (*d*) Example of intensities collected on the XPAD 2D detector during a 2Θ scan of the detector arm: they highlight the diffraction signal filtered by this single-crystal-comb with an angular pitch of 0.1° between each Si(111) crystal. (*d*′) Definition of the small reception windows on the detector for three analyser crystals (Nos. 1, 6, 13) with an example of the intensities collected with a 0.002° step.

In order to greatly improve the regularity and precision of the inclinations of the successive planes of the Si(111) analysers, *in fine*, we realize a multi-analyser comb directly from a single large silicon crystal. This crystalline comb of Si(111) planes was cut from a single large silicon crystal which allows identical and strictly parallel Si(111) analyser crystal blades to be generated perpendicularly [Fig. 8 ▸(*a*)]. Between these Si(111) planes we have cut gaps which allow the diffracted beams to pass. In addition to these slits under all crystals, the single-crystal-comb contains a thin link between them which allows the stresses in the crystal plates to be released when the comb is bent to give the desired LogSpiral curvature. For this prototype, designed to be placed at a distance *D* = 973 mm from the sample, the logarithmic spiral curvature was also optimized to operate over an energy range of 22–46 keV, with a very small pitch (0.1°) between each crystal in order to test the case of a compact system having a very high density of crystals. The gap between adjacent Si(111) blades is 1.7 mm (thickness 0.5 mm + gap 1.2 mm) to allow the passage and filtering of a 1.0 mm-wide beam at an energy of 22 keV close to the energy of an Ag *K*α_1_ emission source. The chosen length of each Si(111) single crystal formed by this comb is 12.6 mm and allows the filtering of a 0.55 mm-wide beam at an energy of 46 keV. The cutting/polishing of such a single-crystal-comb block, whose dimensions are 50 mm × 13.7 mm × 106 mm, is the most delicate operating step and it generates for each Si(111) crystal a dimension of 20 mm × 12.6 mm × 0.5 mm; we thank the ESRF optical department for the precision of this achievement (X-ray Optics ESRF, https://www.esrf.fr/Instrumentation/xray-optics). This single-crystal silicon comb can generate 50 Si(111) blades with a relatively thin bonding framework (0.7 mm) which allows it to accept a 5° LogSpiral bending without generating stresses in the Si(111) crystals and thus to maintain a good resolution for filtering.

In order to bend this single-crystal-comb block, we have produced by 3D printing a rigid polymer support with a LogSpiral curvature corresponding to the previously optimized parameters. This 3D printed holder was made using a multi-jet printing apparatus – ProJet MJP 3600. Although small in size (110 mm long), this block contains a small fixing/stiffening system that allows the curvature of the single-crystal-comb to be optimally adjusted to the LogSpiral curvature of the support [Fig. 8 ▸(*b*)]. The curved single-crystal-comb block is then mounted with its central/middle analyser crystal perfectly aligned on the Θ_A_ axis of the analyser arm, allowing its Θ_A_ orientation to be adjusted to match the energy of the experiment.

This curved single-crystal-comb block is then associated with a Soller-collimator block containing 51 blades having the same angular step (0.1°). This Soller block is also made by 3D printing; its polymer support fixes long (60 mm) absorbing tungsten blades (30 mm × 60 mm × 0.7 mm) with a pitch of 1.82 to 1.88 mm [Fig. 8 ▸(*b*′)]. This Soller block is mounted with a goniometer head on the 2Θ_A_ axis before the 2D detector; the two small vertical/horizontal translations/rotations allow its position to be adjusted perfectly to associate the path of each filtered beam with one collimator. For this adjustment process we used images of the filtered beams on the 2D screen of the detector to check the quality of their alignments independently of the 2θ angle of the detector. A very good accuracy of the crystals alignment can thus be achieved, allowing the generation of a comb of a perfect single-crystal comb with a pitch of 0.1° (±0.01°). This fine tuning only needs to be done once during the energy changes of the X-ray source. For the two extreme energies 22 keV and 46 keV of the usable range, these two blocks are the same – only the angles Θ_A_ and 2Θ_A_ vary (Θ_A_ = 5.1556° at 22 keV and Θ_A_ = 2.4632° at 46 keV). Fig. 8 ▸(*c*) simulates the beam path at 46 keV for such a system using these two rigid blocks: the compact-rigid MAD block contains 50 Si (111) single-crystal blades and the associated Soller-collimator block contains 51 long collimator blades (60 mm).

We tested this single-crystal-comb block associated with a Soller-collimator block by collecting diffraction reflections of a reference LaB_6_ powder at an energy of 22 keV on the D2AM beamline at the ESRF (Ferrer *et al.*, 1998 ▸; Basolo *et al.*, 2007 ▸; Chahine *et al.*, 2021 ▸). For this experiment, performed with a quasi-parallel beam (0.4 mrad), the same large distance (*D* = 973 mm) between the sample and the single-crystal-comb was used and the Soller-collimator block was aligned on arm 2Θ_A_ at a distance (*D*_s_ = 1033–1093 mm) from the sample. The 2D detector used was an XPAD S70 hybrid pixel photon-counting detector (Basolo *et al.*, 2007 ▸; Medjoubi *et al.*, 2010 ▸) which consists of a succession of chips each containing 80 × 120 hybrid pixels. The dimension of each pixel is 0.125 mm which, for the used sample-to-detector distance (1200 mm), allows an angular accuracy of 0.006° per pixel. The data collection of the whole diffractogram of the LaB_6_ powder confirmed the importance of an additional shielding with a simple lead sheet, to protect the beam paths between the single-crystal-comb, the Soller-collimator block and the 2D photon-counting detector, to take full advantage of the background reduction provided by the diffraction filtering. Due to some constraints and fixing limits of the diffractometer arm during this experiment, the distance between the Soller-collimator and the 2D detector was quite large, so this drawback did not allow us to use the entire angular range of the MAD+Soller system and of the detector window; only the first four detector chips were accessible [Fig. 8 ▸(*d*)]. As shown in this figure, the very good angular alignment (±0.01°) of the Si(111) blades of the single-crystal-comb allows a very good separation of the filtered beams with an angular pitch of 0.1°. These images collected on the 2D detector facilitate the positions/orientations alignment of the of the MAD block and the Soller-collimator block before fixing them.

**Figure 9:**
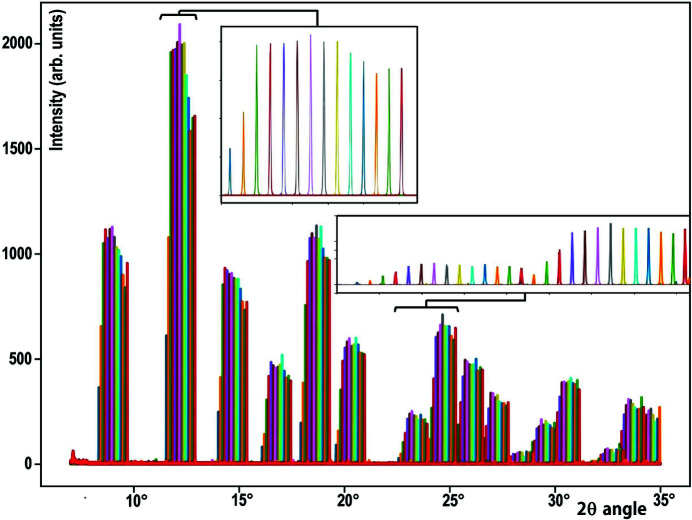
Diffraction patterns of a LaB_6_ sample filtered by each analyser crystal of the Si(111) single-crystal-comb MAD block associated with the Soller-colimator block (one colour for each analyser). The two enlargements detail the signals received for three LaB_6_ reflections after their filtering by each 14 analyser crystals with an angular pitch of 0.1°.

During the powder diffraction measurement, a simple scan of the 2Θ arm drives both the single-crystal-comb + Soller-collimator blocks and the 2D detector, and then integration of the 2D detector data is performed to obtain a 1D diffraction pattern versus the 2Θ angle (Basolo *et al.*, 2007 ▸; Dejoie *et al.*, 2018 ▸; Fitch & Dejoie, 2021 ▸). The measurement of the LaB_6_ diffractogram and the selection of the signal filtered by each crystal of the MAD analysers is obtained by defining for each filtered beam a very small number of pixels receiving the appropriate signal [Fig. 8 ▸(*d*′)]; their small size allows an optimization of the signal-to-noise ratio of the measurements. We can then collect the LaB_6_ diffraction pattern, identifying if necessary the rays coming from each Si(111) analyser crystal (Fig. 9 ▸).

## 4. Conclusion

The main interest of all these MAD filtering systems is that they can be associated with 1D–2D detectors to collect accurate powder diffraction data more quickly. Their common key point is the layout of their multiple diffraction analysers located on a logarithmic spiral curve, which allows parallel diffraction filtering of different beams arriving on the 1D–2D detector. The three MAD filter configurations [(*a*), (*b*), (*c*)] are dedicated to X-ray analyses of complex samples with good resolution and low instrumental noise. For high-resolution diffraction/scattering experiments these MAD filters allow a good filtering that fully complements the qualities of the new photon-counting detectors which have very low intrinsic noise. Their combined use makes it possible to drastically overcome the current limits of weak signals detection necessary for the detection of minor phases in studies of ‘real’ heterogeneous materials (*e.g.* pigments in paints, inclusions in alloys, ‘dirty’ powders, powders on mixed supports, *etc.*). Indeed, thanks to this MAD multi-filtering, we can improve the detection limits from 3–1% to 0.1% and also improve the accuracy of the selection of individual signals of the sample under analysis. In this Part 1 contribution, we have presented several possibilities to realize them, by using simple systems, in order to obtain a notable gain for experiments carried out in a SR facility but also in the laboratory with a classical X-ray source.

The possibilities of the filtering configuration (*a*) are more dedicated to experiments using low-energy X-rays (*e.g.* Cu *K*α_1_ in the laboratory) which can be used for analyses of the object in reflection mode (*e.g.* heritage statue, large piece of metallurgy, powders, thick support,…). The possibilities of configurations (*b*) in Laue mode by transmission and (*c*) in Bragg mode by reflection are more dedicated to experiments using high-energy X-rays (*e.g.* Ag *K*α_1_ or Mo *K*α_1_ in the laboratory and high energy in a synchrotron radiation facility), which can be used for analyses in transmission mode on the object (*e.g.* very small sampling of culture heritage object, painting, metallurgical sheet, powders, thin support,…).

We have optimized the configuration (*c*) by making a MAD single-crystal-comb block curved on a logarithmic spiral support surface. This small rigid system allows efficiency gains associated with an excellent filtering and a very high resolution over a wide range of synchrotron X-ray energies. The chosen geometries and energies, as well as the small size of the single-crystal-comb and Soller-collimator blocks, show that such a MAD filter system could also be used to increase the resolution of experiments using laboratory X-rays sources. Our first tests measured on a LaB_6_ reference sample are very promising. These results are confirmed by the complete data collections of powder diffractograms of a LaB_6_ reference sample and also of several complex samples, performed on the D2AM beamline of the ESRF at an energy (22 keV) close to that of a laboratory Ag *K*α_1_ source and reported in the associated contribution Part 2 (Hodeau *et al.*, 2023 ▸).
